# On the Nature and Treatment of Miasmatic Fevers

**Published:** 1846-02

**Authors:** George L. Upshur

**Affiliations:** Norfolk, Va.


### MEDICAL EXAMINER,

AND

### RECORD OF MEDICAL SCIENCE.

NEW SERIES.—No. XIV.—FEBRUARY, 1846.

### ORIGINAL COMMUNICATIONS.

*On the Nature and Treatment of Miasmatic Fevers.* By GEORGE L. UPSHUR, M. D.

Upon no subject within the range of medical science, has so much been written, or so many hypotheses been promulgated, as the nature of the agent which produces miasmatic fevers. While all agree that they have their origin in the exhalations from marshy lands, and decaying vegetable matter, no one has yet been able to isolate the subtle poison, or satisfactorily account for the phenomena of its action upon the system. A vacuum is thus left in the description of a train of diseases, whose unmanageable nature and frequent fatal terminations have gone far towards placing them among the *opprobria medicorum.* We should, therefore, give quiet heed to any hint which is at all calculated to throw light upon a subject which is as important as it is obscure.

In the *Journal des Connaissances Medico Chirurgicales,* for September, 1845, there is a well written article from the pen of M. Berard, styled, "What is the nature of the miasm producing Intermittent Fever?" The author there contends, that miasmatic fevers owe their origin to the introduction of hydrogen gas into the system. As the theory is novel and ingeniously sustained, I shall be pardoned for giving it here in the words of its author:

"The human mind is so constituted, that it seizes upon hypothesis the moment that truth fails it. We know it is said, the effect of nitrogen, of oxygen, and of carbonic acid upon the system; that is true, since these gasses all concur to form atmospheric air. We know, moreover, the effect of hydrogen gas upon the respiratory function; that it can be breathed without danger; and it is unnecessary to state the conviction, that, up to this moment, no one has ever attributed the phenomena of an attack of intermittent fever to the introduction of hydrogen gas into the air passages. M. Desroches says, however, that if hydrogen gas is not hurtful of itself, it becomes so by displacing oxygen. But what, let me ask, would be the effect, if a large portion of this gas, whether by absorption or otherwise, should gradually penetrate into the circulation, and suddenly displacing oxygen, combine with it? I defy any one to follow this proposition through all its developments without the history of the three stages of intermittent fever. Let us try:

"*First stage of the fever.* Retrocession of blood from the extremities to the centre; horripilation; chills; shivering. The hydrogen introduced into the circulation displaces oxygen, for which it has an affinity greater than for any other body. The combustion of carbon by oxygen in the lungs—*the principal source of animal heat*—gradually ceases, and general cold follows.

"*Second stage.* The slow and progressive development of heat; it flows, at first, from the great circulatory centres, where caloric is naturally preserved; all parts of the body are soon invaded by it, and the patient soon finds himself actually in a state of ignition. Hydrogen, once combined with oxygen, enters into a state of fermentation; this effect is in proportion to the quantity of heat that remains. Soon, however, the disengagement of caloric advances to a considerable point.

"*Third stage.* The skin, which was very hot, now becomes moist; the sweat is fully established, and it flows sometimes in an inexplicable abundance. Having arrived at the highest point of effervescence and combustion, the hydrogen and oxygen unite and resolve themselves into water.

"Here then, if I mistake not, are the cold, the hot, and the sweating stages of intermittent fever.

"If we examine, now, this theory by the multitude of facts which are known to us, we shall be struck by their evidence. Thus, whether we consider marshy districts as immense reservoirs of hydrogen, and consequently as perpetual seats of infection; or, on the contrary, whether we look at the forests and woodlands where intermittent fever is unknown—*because, we say, hydrogen is there constantly absorbed by the vegetation*—either side agrees with our ideas.

"The state of the atmosphere and the seasons will furnish us with other proofs not less conclusive. I say further, that on inquiring into their influence upon this disease, and in seeking the part which they take in its development, we shall be forced to the conclusion, that intermittent fever is never found without the concurrence of hydrogen gas.

"It is not strictly true, however, that atmospheric vicissitudes are *alone* sufficient to engender intermittent fever. If that was so, the spring, which at Rome is, beyond contradiction, the most variable season, and most subject to violent changes of weather, would be, *par excellence,* the season for fevers; but so far from that, fevers are rare at that time; and when, by chance, you find a few cases of the past autumn continuing during winter, you see them, in the spring, going away of themselves without treatment; disappearing, according to a popular saying, at the appearance of the first cherries. That is to say, when the rapid advance of the young vegetation absorbs from the hurried vapors of the air every thing which contains hydrogen.

"There is one thing, however, about which there can be no doubt, namely, that during any season in Rome, the approach of a storm is sufficient to induce an attack of fever in a number of persons. I beg you to observe, that I say only the *approach* of a storm, for then the air is charged with electric fluid, and a sudden diminution in the pressure of the atmosphere sets free a large quantity of gas from the marshes; as soon, however, as it combines with the surrounding oxygen, and the rain begins to fall in torrents, all danger is over! Any one, who has ever been to Rome, has heard this saying from the mouth of almost every person, that heavy rains chase away the fever.

"A similar result follows long droughts,—two atmospheric conditions which doubtless seem opposed, but experience confirms the truth of the assertion. There are no cases of intermittent fever at Rome during the great summer heats, and that is explained by the rapid absorption of the slightest exhalations, and by the weight of the atmosphere which maintains the gas at the bottom of the water by suspending all fermentation.

"Summer, then, with its heats, without the possible production of hydrogen; winter, with its abundant rains, which dissipate this gas entirely; and spring, with its vicissitudes, hastening the vegetation which absorbs it, have no cases of intermittent fever. Autumn alone, which is attended by none of these conditions—which has neither excessive heats, abundant rains, nor vegetation—is devoted to all the severities of the epidemic. But how does this take place? The soil is burnt, and deprived of its products; the marshy lands are dry; and every thing, deprived of life, lies parched upon the surface of the earth. At this time a slight rain falls, a little humidity penetrates the soil; every thing then begins to ferment—the decomposition of animal and vegetable substances begins, and torrents of hydrogen gas, *which there is nothing to absorb*, are set free, and fill the air to prepare the cataracts of the season which are to follow."

I have given a literal translation of enough of M. Berard's communication, to show upon what principles he bases the idea, that miasmatic fevers owe their origin to the introduction of hydrogen into the system. His theory, though somewhat fanciful, is, nevertheless, both plausible and captivating, and he urges it upon our attention with all the zeal of one wedded to a new hypothesis. We should not suffer ourselves, however, to be drawn from the truth by freaks of the imagination, but should ever keep in view the fact, that theory, no matter how ingenious and pleasing, is utterly worthless unless based upon well established laws. While, therefore, I do not desire to detract from the credit to which M. Berard is justly entitled, for his endeavors to discover the true nature of the cause producing miasmatic fevers, it will not be deemed amiss to present some valid objections to his theory. I object to it—

1st. Because it bases the cold stage of fever upon a statement, which is not in accordance with the views of more enlightened physiologists, namely, that animal heat is generated by the union of oxygen and carbon, *in the lungs only*. Liebig contends, that though the source of animal heat is attributable to the combustion of carbon, this combustion occurs not only in the lungs, but in the capillary system of the entire body, and he supports his hypothesis by a train of reasoning, founded upon experiment, which has never yet been overturned. Again, it has been pertinently asked, if the combustion of carbon takes place in the lungs, generating a heat in these organs which is subsequently carried through the whole frame, why is it that the parts within, and adjacent to the thorax, have not a higher temperature than other parts of the body? An inevitable conclusion, but one which is not upheld by actual experiment, since blood taken from the lower extremities is found to be of the same temperature as that drawn from the carotid artery.

2d. Because it is clearly deducible from his theory, that the cold and hot stages must always be in the same ratio, as regards their duration, instead of in an *inverse* ratio, as is known to be the case! For, if the cold of the first stage is produced by the introduction of hydrogen, the greater the quantity of gas present the longer the first stage; and if the second stage be caused by the combustion of hydrogen, the larger the amount of the *fuel* the longer will be the hot stage. All experience, however, proves that the longer the first stage of intermittent, the *shorter* the second, and vice versâ.

3d. The theory is not valid, because it fails to account for the fact, that in some cases of intermittent fever *there is no hot stage between the cold and the sweating stages.* We frequently meet with cases where reaction is slow in making its appearance; the patient remains for hours in a shivering condition, with the surface pale and cold, while the perspiration, cold and clammy, issues in large drops from every pore, constituting the *pernicious intermittent* of the French, and the congestive fever of other authors. Surely, in such a case, it can not be said, that the sweat is produced by the combustion of hydrogen with oxygen, for such combustion can not occur without the disengagement of a considerable quantity of caloric.

4th. I object to the theory, because it can not account for the subsequent paroxysms. Thus, a person inhales to-day a portion of hydrogen—it prevents the union in the lungs of a portion of oxygen with carbon, and general cold follows, which is the first stage of the fever. Soon the hydrogen enters into a state of combustion with the displaced oxygen, and heat is the result, which forms the second stage. Having reached the highest point of combustion, water is formed, which constitutes the third, or sweating stage. As soon as the perspiration begins, the patient feels relieved—in a little while, his appetite returns, and he is apparently free from disease. On the next day, however, or the day after, the same symptoms return, run the same course, and are again followed by an interval of apparent health. This state of things may exist for weeks or months; even continuing, according to M. Bérard himself, throughout the winter—a season during which all the hydrogen which is generated is dissipated by heavy rains. If hydrogen acts to produce intermittent fever, in the manner contended for by the author of the theory which we are considering, it must require a fresh inhalation of the gas just prior to the approach of each paroxysm, and it is extremely improbable in some cases, and impossible in others, that such inhalation takes place.

5th. I object to the theory, because experience proves that those persons who are most frequently exposed to an atmosphere charged with hydrogen gas, such as lecturers on chemistry and their pupils, æronauts and some others, are not more subject to attacks of intermittent fever than other persons.

6th. Because hydrogen gas, being lighter than all other bodies, will rise high above the surface of the earth, while it is proved that, in miasmatic regions, those who live in the lower stories of houses are much more subject to attacks of fever than those who occupy the higher apartments. Dr. Magill says, "The first characteristic which strikes us in regard to malaria, is its *superior density*, when contrasted with atmospheric air. The fact of the greater exemption from disease of those who live in the upper stories of houses where miasmata abound, is well known to those who have paid any attention to the subject. Dr. Furguson (Phila. Journal, No. 13, p. 18,) states, that 'according to the official returns, during the last sickly season at Barbadoes, the proportion of those taken ill with fever in the lower apartments of the barracks exceeded that of the upper by one-third, throughout the whole course of the epidemic.'"*

7th. M. Bérard's hypothesis does not account for the phenomena of *remittent* fever, a disease which undoubtedly is due to the influence of miasma. Thus, there is frequently no sweat throughout the course of remittent fever, the disease often running its course, *with the skin dry*, and having a favorable crisis in copious hemorrhage from the nose or pharynx, or by a discharge of thick, offensive matter from the bowels.

8th, and last. This theory cannot explain why there is a number of affections as constantly found in miasmatic regions as is intermittent fever, but which do not at all resemble this last in any respect—such are many forms of neuralgia, chlorosis, apoplexy, palsy, visceral obstructions, dysentery, diarrhœa, and some others.

From these remarks it will be perceived, that M. Bérard's theory, ingenious though it be, is opposed by objections so forcible, that we are left as much in the dark in regard to the nature of the miasm producing intermittent fever, as though his theory had never been published.

It is to be feared, too, that we are destined long to remain ignorant upon this important subject, and that marsh miasmata, like the subtle poison of contagion, will ever be known, as heretofore, only by its calamitous effects.

There is cause for gratulation, however, that although we cannot analyse the morbific agent, its effects upon the system have been so carefully studied as to lead to a rational mode of relieving them. To this poison of the subject I desire to turn my attention before bringing this communication to a close.

In the milder forms of *intermittent* fever it is a common and very successful practice, among the physicians of lower Virginia, to exhibit at the onset of the cold stage an emetic and cathartic combined. The act of vomiting tends to remove congestion of internal vessels and organs, and, by forcing the blood to flow with greater rapidity through its channels, to disturb in a great degree, the morbid action in the system; while the cathartic,

* Lectures on Malaria, by A. T. Magill M. D. 1836—p. 6.

which usually moves the bowels towards the conclusion of the hot stage, promotes perspiration, and prepares the frame for the reception of medicines which are proper to prevent a second paroxysm. The emeto-cathartic is usually composed of calomel gr. x., tart. emet. gr. ij.

*Opium* is highly recommended by Drs. Shapter, Elliotson, and other writers, as a good remedy in the cold stage. According to my own experience, however, it is apt to hasten congestion of the liver and brain; to render the bowels still more costive, and thus to bring on a comatose state from which the patient seldom rallies. Especially do these effects follow the exhibition of this drug in cases of long duration of the cold stage, with great depression of the vital powers, constituting the *pernicious intermittent.*

*Blood-letting*, in this stage, I have seen of great service, and have no hesitation in subscribing to all the praises bestowed upon it by Dr. Mackintosh and other authors; with this restriction, however, that it shall be resorted to only in cases where the patient has not been much debilitated by previous disease.

The distressing nausea which is often attendant upon the first stage of intermittents, I have known promptly relieved by large draughts of the *cold* infusion of the *thoroughwort*, (Eupatorium perfoliatum.) This plant, indigenous to the more temperate parts of the United States, and easily procured by all who seek for it, has been too much neglected by the profession. Apart from its tonic and diaphoretic properties, which are undoubted, it possesses the peculiar power of allaying restlessness, and relieving the distressing aching of the limbs and joints so generally found in ague. It is a very common domestic remedy in this section of country, though not often prescribed by physicians; and many cases of simple intermittent are entirely cured by it, without a resort to any other remedy. From my observation of its action, I have no doubt but that it possesses a principle but little inferior to quinia in its anti-periodic powers.

During the hot stage, the patient should be kept moderately covered, and with a free circulation of air through the room. To allay the urgent thirst, ice finely powdered, and exhibited in teaspoonful doses every ten minutes, or oftener, will be found useful. The sweet spirits of nitre, and, according to Dr. Graves, the infusion of cascarilla acidulated with the elixir of vitriol, will also be beneficial. Should there be much fulness of the head, with burning of the eyes and injection of the conjunctiva, it will be proper to take a few ounces of blood from the arm.

I have frequently seen these symptoms, however, completely subdued by the following mixture:

℞. Potass. Nitrat. ℈ ij.
Tart. Emet. gr. ij.
Spts. Nit. Dulc. f. ℥j.
Aquæ fontis f. ℥iij.
M. S. a tablespoonful every hour.

As soon as the hot stage begins to decline, which is evinced by the appearance of slight moisture in the palms of the hands, we should commence with the exhibition of the *sulphate of quinine.* To wait until there is a complete cessation of febrile action, is to lose time and thereby hazard the recurrence of a second paroxysm. This remedy is undoubtedly of the first importance in the treatment of all miasmatic fevers, whether they be of an intermittent or remittent character. Most systematic writers upon this subject, however, direct quinine to be given in cases of *intermittent*, in doses, and at intervals, proportioned to the type of the disease; while in *remittent* there is scarcely one who does not condemn its use *in toto.* Thus, in the *quotidian*, we are told to administer two grains every hour until fifteen grains have been given; that in the *tertian*, one grain every hour or two, until the same quantity has been given, will prevent another paroxysm; while in the *quartan*, it may be exhibited in smaller doses at even longer intervals. (*Shapter*, *Brown*, *Watson*, *Eberle*, *Elliotson and others.*) As it is impossible, however, when called to a patient just seized with fever, to determine what type it will assume, and as it is frequently of vital importance to ward off a second paroxysm, this mode of administering quinine will often fail to accomplish the desired effect. My invariable practice is, as soon as there is the slightest diminution in the intensity of the hot stage, to exhibit quinine in doses of three or five grains every two hours, *until the patient complains of noises in his ears.* It has long been known, that cinchona and its preparations, when exhibited in considerable doses, will produce such noises in the ears as buzzing, ringing, roaring, &c., but I am not aware that it has heretofore been proposed *to consider such effects as demonstrative of the entire subjection of the system to the influence of this remedy.* It is to this point, then, as one of considerable importance in the management of miasmatic fevers, that I wish particularly to direct attention.

In the exhibition of *mercurials*, when it is desired to produce their specific effect upon the system, their use is discontinued upon the appearance of *ptyalism*, since it is well known that if benefit does not result when the mouth becomes sore, no good is to be expected from a continuance of the remedy. So, too, in using *arsenical preparations,* we look for no further benefit from them after *œdema* and *gastric disturbance* have supervened; upon a principle similar to these, I give the sulphate of quinine in miasmatic fevers. As soon as ringing, buzzing, or roaring in the ears come on, I feel satisfied that the medicine has produced its strongest impression, and that its further exhibition would be worse than useless. *If the disease do not then yield, it must be combatted with some other remedy.* Sometimes I have seen only four grains produce this effect—often it will require forty grains, and in some rare cases, doses incredibly large, will fail to accomplish it. Cases of the last description, according to my observation, terminate fatally; *while, on the other hand, I have never lost a patient after quinine had affected the head, in the manner above indicated.*

I am well aware that many a case of miasmatic fever terminates favorably, though quinine has not been used to the extent here recommended; but I know, too, that many a case continues for weeks which might have been cured in forty-eight hours. The action of quinine upon the system is perfectly incompatible with that of malaria—therefore, if it require but a *single grain* to render harmless the poison, give it; if it require *a drachm,* do not hesitate to give it; and whether it will require a grain, a drachm, or any quantity greater or less than either of these, may be ascertained by reference to the effect produced upon the patient's head.

Since March, 1844, I have treated ninety cases of intermittent, and fifty-eight cases of remittent fever, in all of which quinine was exhibited until the head felt its effects. All recovered; and in no instance did harm result from this manner of giving the medicine. In a few cases, the patients were disposed to be restless, but a simple purgative, and one night's sleep were sufficient to carry off every unpleasant symptom. Nor in intermittent fever, have I ever seen a recurrence of the paroxysm; or in remittent the continuance of febrile action for more than eight hours, *after ringing, roaring, or buzzing in the ears supervened.* Instead, therefore, of recommending quinine in doses, and at intervals, according to the type of the fever, I would, in every instance, give it in large doses every hour, until the patient complains of peculiar sounds in his ears, as above indicated. And I as invariably consider my patients out of danger when *tinnitus aurium* comes on during the exhibition of quinine in miasmatic fevers, as I do when *ptyalism* supervenes after the exhibition of mercurials in any of the phlegmasia.

Dr. W. J. Tuck, of Memphis, has recently published in the *New Orleans Medical and Surgical Journal,* a very interesting paper upon the employment of large doses of quinine in bilious remittent fever. As far as my experience goes, it is perfectly in accordance with that of Dr. Tuck. We do not often find, in this section of country, remittent fevers characterised by such malignant symptoms as are attendant upon them in the West and South-west, and, therefore, it is seldom necessary here to give quinine in such large doses. But, as far as the propriety of exhibiting it, *while the fever is on*, is concerned, I think there is no question. I have given it in ten grain doses, and frequently in doses of five grains every hour, with such marked benefit as to leave no doubt on my mind, that it is not only the best, but in some cases the only efficient treatment. There is, unfortunately, a great prejudice in the profession against what many call "heroic" doses of quinine, in the treatment of marsh fevers—a prejudice, which enlarged experience in the management of these affections can alone remove.

Since the time of the celebrated Radcliffe, the free use of *mercurials* in miasmatic fevers has been an almost universal practice. While, therefore, I am far from desiring to discard, altogether, a remedy whose beneficial use is attested by many distinguished men, I cannot but believe that in intermittent and remittent fevers, *uncomplicated with inflammation*, mercury is too often given to an unnecessary and injurious extent. In this section of country, a majority of physicians have an unconquerable dislike to giving quinine, except during complete apyrexia, and with such the practice of treating fevers with mercury, until ptyalism supervenes, is extremely common. This practice is unphilosophical, unless those who pursue it hold to the homœopathic motto, *similia semilibus curantur*, for the morbific agent which engenders miasmatic fevers, and mercurials, produce the same pathological alteration of the blood—*they render its fibrine less abundant, and thus destroy its coagulability.* To give mercury, then, in idiopathic fever, is but to hasten the end to which the fever naturally tends.

I know that mercurials, unassisted by the other medicines, have the power of putting an end to the course of miasmatic fever, for I have experienced this in my own person; but the question arises, is not the patient left, after such a cure, (?) in a condition equally dangerous, as that produced by the fever itself? The practice may well be likened to the putting out the fire in a burning house by forcibly tearing the house in pieces; it is left as unfit for a tenant as if the flames had been permitted to destroy it unmolested. This view is fully confirmed by observation. The physicians of Northampton county, Virginia, annually attend a vast number of cases of miasmatic fever, and their adherence to the mercurial practice is extremely rigid. The country there is flat, and the sources of malaria abundant.

The fevers, however, though so prevalent that during some sickly seasons scarcely an inhabitant escapes an attack, are seldom complicated by visceral disorder. There are, consequently, few fatal cases. Great prevalence of fever, however,—no matter how mild in its character—during the autumnal months, is sure to be followed by great mortality during the following winter, from typhoid affections of the brain, lungs, and chylopoietic viscera. I attribute this to the almost exclusive use of mercurials in the management of the autumnal fevers. Such a practice tends to defibrinise the blood, to destroy in a great measure its power to coagulate, and thus to render it so fluid that it flows with difficulty through the capillary vessels. Hence, persons who have an attack of intermittent, or remittent fever, during the autumn, and take for its relief large quantities of mercury, find themselves much enfeebled, anæmic, and easily affected by even the slightest exposure. It is no wonder, then, that as the inclemencies of winter approach, such persons should be so little able to resist sudden congestion of the different organs, which rapidly tends to a fatal termination. In Northampton, the autumn of 1842 was characterised by unusual prevalence of fevers, though there was but little mortality attendant upon them. During the ensuing winter, however, there were considerably more than 100 deaths, the population of the county being about 7000. Persons were frequently destroyed in eight or ten hours, from violent congestion of the lungs or brain; and in those cases which lingered for a longer period, there was such a loss of vital power as to call for the exhibition of the most powerful stimulants. The most quickly fatal cases were those in which salivation had been produced during the previous autumn.

In this city, where we rely more upon quinine than mercurials in intermittent and remittent fevers, our winters are comparatively free from inflammations of a typhoid character. I do not intend, by the foregoing remarks, to be understood as condemning mercury altogether in fever. Given in the commencement of the disease, with a view to its cathartic properties; and, in fever complicated with inflammation, with a view to its specific impression, it is very serviceable. It is only against the routine practiceof prescribing mercury in every case of miasmatic fever, whether it be simple or compound, mild or severe in its symptoms, that I contend. The physician who pursues a routine practice, and treats disease solely from its name, is certain to be an empiric.

There is a variety of miasmatic fever, occurring chiefly among children between two and seven years, which is characterised by shorter remissions, and more gastric irritation, than is usually found in the more idiopathic form of the disease, and which is sometimes extremely difficult to manage. The appetite is whimsical, the tongue pointed and red at the edges; the eyes have a watery, heavy look; the bowels are frequently loose, the dejections being yellow, and sometimes brownish and offensive. There is a well marked remission every morning, though its duration is brief; and the patient is restless and irritable during the fever. The thirst is not very great, nor is nausea very common. There is more or less tympanitis, especially just prior to the exacerbation of fever. In the management of such cases, I have found quinine very offensive to the stomach, being rejected as soon as it is swallowed. This effect follows even if the taste of the medicine be entirely disguised, by giving it in the form of pill. When retained, however, it will often put a stop to the symptoms for a day or two, when they will almost invariably return. I have found the *ol. terebinthinæ* in combination with the *hydrargyrum cum cretâ* very serviceable in this form of fever. It is given thus:

℞. Hydrarg. c. cretâ ʒj.

Ol. terebinth. f. ʒj.

Acaciæ gr. xxx

Aquæ f. ℥ij.

M. S. A teaspoonful every two hours.

In some cases, where every remedy had failed, the application of a blister to the epigastrium produced a complete cure.

I have said nothing in regard to the management of complications occurring during the course of miasmatic fevers, because this communication has already been extended to too great a length. I cannot conclude, however, before urging upon members of the profession, whose experience is great, to give to the medical world their views upon the management of this class of diseases. It is a field which, though frequently travelled over, has been examined minutely by only a few. Captivating theories have unfortunately usurped the place of practical observation and well founded facts. No medical man should pass through life without having profited by the lesson taught in the remark of the great Sydenham: "But how great soever others' endeavours have been, I always thought I lived in vain, unless I, being of the same employment, contribute something, how small soever, to the treasury of physic."

*Norfolk, Va.*, Dec. 15, 1845.

